# Hyperspectral Imaging and Interpretable Machine Learning for Bleaching-Level Identification and Treatment-Associated SO_2_ Residue Estimation in Lanzhou Lily

**DOI:** 10.3390/foods15203650

**Published:** 2026-10-14

**Authors:** Yueling Yan, Maomei Xie, Jinzhi Liu, Liyi Deng, Jie Qiu, Shuang Yang, Lei Nie, Zheng Li, Huaxin Zhou, Haixia Wang

**Affiliations:** 1 College of Pharmaceutical Engineering of Traditional Chinese Medicine, Tianjin University of Traditional Chinese Medicine, Tianjin 301617, China; y3178002270@gmail.com (Y.Y.);; 2 NMPA Key Laboratory for Technology Research and Evaluation of Drug Products, School of Pharmaceutical Sciences, Cheeloo College of Medicine, Shandong University, Jinan 250012, China; 3 Tianjin Key Laboratory of Intelligent and Green Pharmaceuticals for Traditional Chinese Medicine, Tianjin 301617, China; 4 Haihe Laboratory of Modern Chinese Medicine, Tianjin 301617, China; 5 State Key Laboratory of Chinese Medicine Modernization, Tianjin 301617, China

**Keywords:** hyperspectral imaging, interpretable machine learning, SHAP, SO_2_ residue, identification and prediction, Lanzhou lily

## Abstract

Rapid and nondestructive assessment of sodium metabisulfite (Na_2_S_2_O_5_) bleaching treatments and sulfur dioxide (SO_2_) residues is important for the quality and safety control of Lanzhou lily. This study integrated hyperspectral imaging (HSI), machine learning, and SHapley Additive exPlanations (SHAP) to identify bleaching levels and estimate treatment-associated SO_2_ residues. Model performance was assessed using treatment-replicate-grouped validation with samples from a single procurement lot. For bleaching-level classification, the support vector machine (SVM) combined with normalization (NOR), denoted as SVM-NOR, achieved the highest accuracy of 0.902. For SO_2_ residue estimation, the random forest (RF) model using raw spectra, denoted as RF-Raw, provided the best performance, with a cross-validated coefficient of determination (R^2^) of 0.978 and a root mean square error (RMSE) of 38.5 mg/kg. The reported regression performance reflects treatment levels represented in calibration. Complementary leave-one-concentration-out (LOCO) validation yielded a higher pooled RMSE of 155.4 mg/kg for held-out levels. SHAP analysis revealed different wavelength-contribution patterns for classification and regression, providing an interpretable basis for the model predictions. These findings suggest the potential of HSI for rapid prescreening, while further validation is needed for predictions between calibrated treatment levels, particularly near the regulatory reference.

## 1. Introduction

Lanzhou lily is a well-known regional specialty crop in China. It is highly valued for its fleshy white scales, delicate texture, sweet flavor, high nutritional value, and dual role as both a food and medicinal resource. Consequently, it is widely used for fresh consumption, food processing, and traditional Chinese medicine [1,2]. However, the high moisture content and fragile cellular structure of Lanzhou lily scales make them highly susceptible to enzymatic browning during postharvest operations, such as grading, washing, slicing, drying, and storage. Consequently, the scales exhibit surface discoloration, reduced brightness, and impaired color uniformity, ultimately decreasing their commercial value and consumer acceptance [3,4]. As these appearance changes may continue and intensify during subsequent storage, rapid monitoring of color deterioration is essential for assessing the quality of stored Lanzhou lily products and supporting effective postharvest management. Color is the primary quality attribute influencing consumer acceptance of Lanzhou lily and plays a crucial role in its marketability, processing value, and brand competitiveness. To enhance product appearance and suppress enzymatic browning, sulfur-containing bleaching agents, particularly sodium metabisulfite (Na_2_S_2_O_5_), are commonly applied during postharvest processing [5]. In aqueous solution, Na_2_S_2_O_5_ releases sulfur dioxide (SO_2_) and sulfite species, which effectively retard enzymatic browning in lily scales by inhibiting polyphenol oxidase (PPO) activity, reducing quinone intermediates, and scavenging reactive oxygen species [6]. Appropriate Na_2_S_2_O_5_ treatment can improve the brightness and color uniformity of lily scales. However, excessive or improper application may result in over-bleaching, deterioration of tissue integrity, and undesirable changes in flavor, while simultaneously increasing the risk of excessive SO_2_ residues. Excessive SO_2_ and sulfite residues may cause respiratory irritation and allergic reactions, particularly in susceptible individuals such as patients with asthma [7]. Therefore, the development of a rapid, nondestructive, and accurate method for bleaching identification and SO_2_ residue detection in Lanzhou lily is of considerable importance for ensuring product quality and safety and facilitating quality control during storage, processing, and distribution.

Currently, the detection of SO_2_ and sulfite residues in foods mainly relies on chemical analysis, chromatographic techniques, and spectroscopic methods [8]. Conventional chemical methods, such as the pararosaniline hydrochloride method, iodometric titration, and distillation–titration, offer high sensitivity and well-established standardized procedures, and are therefore widely used for laboratory analysis and food safety monitoring [9]. However, these methods are destructive, require labor-intensive sample pretreatment, consume large amounts of reagents, and are time-consuming, making them unsuitable for rapid on-site screening and nondestructive detection. Instrumental analytical techniques, including high-performance liquid chromatography, ion chromatography, and gas chromatography, further improve analytical accuracy and quantitative sensitivity. Nevertheless, their widespread application in food processing and commercial distribution is limited by the high cost of instrumentation, complex analytical procedures, and the need for specialized personnel and laboratory facilities [8].

In recent years, nondestructive techniques, including near-infrared spectroscopy (NIRS), electronic noses (E-noses), and hyperspectral imaging (HSI), have been widely applied in agricultural product quality assessment and food safety monitoring [10,11,12,13,14]. NIRS enables rapid characterization of changes in the internal chemical composition of samples and has been widely applied in agricultural product composition analysis, adulteration detection, and food additive determination [13,14,15,16]. Previous studies have demonstrated that NIRS combined with chemometric methods can achieve rapid quantitative determination of SO_2_ and bleaching agent residues in foods, showing considerable potential for applications in products such as wine, dried fruits, and starch-based foods [15,16]. E-noses, which simulate the human olfactory system, enable rapid detection of changes in volatile compounds and have shown considerable potential in food quality assessment, spoilage monitoring, and the detection of illegal additives [14]. However, single-point spectral or odor-response information is often insufficient to simultaneously characterize the spatial heterogeneity and surface structural variations of samples [10]. In contrast, HSI simultaneously acquires spectral and spatial information over continuous wavelengths [11], enabling comprehensive characterization of not only chemical composition but also color, texture, and structural attributes [17]. Consequently, HSI has demonstrated unique advantages in fruit and vegetable quality evaluation, disease detection, pesticide residue analysis, and process monitoring [10,11,12].

The effects of Na_2_S_2_O_5_ treatment on lily scales are inherently complex and involve multiple scales of variation. Different treatment concentrations not only alter the surface color and reflectance characteristics of the samples but may also influence tissue structure, moisture status, and light scattering properties, resulting in a nonlinear relationship between spectral responses and SO_2_ residues. In modeling high-dimensional hyperspectral data, conventional linear methods, such as partial least squares regression (PLSR), provide good interpretability and robustness but may exhibit limitations in capturing complex nonlinear relationships [18]. Machine learning algorithms, such as support vector machine (SVM), random forest (RF), and k-nearest neighbor (KNN) [19,20,21], are capable of effectively capturing complex nonlinear patterns in high-dimensional hyperspectral data and have been widely applied in intelligent food quality evaluation and safety inspection [10,11,12,17]. However, complex machine learning models are often regarded as “black boxes” because their decision-making processes lack transparency, limiting their practical application and mechanistic interpretation in food safety analysis [22,23]. SHapley Additive exPlanations (SHAP), a game theory-based interpretability method, quantitatively evaluates the contribution of individual features to model predictions and has been widely applied to explain tree-based, ensemble, and other machine learning models [22,23]. Integrating SHAP into hyperspectral data analysis not only facilitates the identification of key wavelengths contributing to bleaching identification and SO_2_ residue prediction but also provides insights into the spectral basis of model decision-making, thereby improving the interpretability, reliability, and generalizability of the developed models.

Based on the above considerations, this study used Lanzhou lily scales as the research material to establish a batch of samples treated with different concentrations of Na_2_S_2_O_5_ and systematically investigate the effects of bleaching treatment on appearance characteristics and SO_2_ residue levels. HSI was employed to acquire the spectral information of the treated samples, and different spectral preprocessing methods in combination with machine learning algorithms were used to develop classification models for bleaching treatment identification and regression models for SO_2_ residue prediction. Furthermore, SHAP was applied to the optimal models to identify the key wavelengths contributing to model performance and to elucidate their influence on model predictions. This study aims to provide a rapid and nondestructive approach for identifying Na_2_S_2_O_5_ treatment and predicting SO_2_ residues during Lanzhou lily processing, while offering methodological support for the application of hyperspectral imaging combined with explainable machine learning in agricultural product quality and food safety monitoring.

## 2. Materials and Methods

### 2.1. Experimental Materials and Sample Preparation

Fresh Lanzhou lily scales used in this study were purchased from Lanzhou, Gansu Province, China, as a single batch and were selected to ensure samples were of uniform size and color, free from mechanical damage, mold, diseases, and insect infestation. After obtaining, the outer senescent scales were removed, and intact edible scales were retained. The scales were rinsed thoroughly with purified water to remove adhering soil and impurities and then drained before further treatment. To minimize the effects of initial enzymatic browning and tissue heterogeneity on subsequent hyperspectral measurements, a mild blanching pretreatment was performed. The cleaned lily scales were immersed in boiling water for 1 min until they became semi-translucent without obvious tissue softening, followed by immediate cooling and draining. This pretreatment was intended to partially inactivate browning-related enzymes, such as PPO, thereby reducing variations in the initial browning status among samples. In addition, blanching improved tissue uniformity, thereby minimizing the influence of structural heterogeneity on hyperspectral reflectance characteristics. After blanching, the scales were pooled and randomly assigned to seven Na_2_S_2_O_5_ treatment groups. Each treatment group contained three treatment replicates of 20 scales, and each replicate was treated in a separate container.

Analytical-grade Na_2_S_2_O_5_ was dissolved in distilled water to prepare treatment solutions at concentrations of 0% (distilled water as the control), 0.3%, 0.5%, 0.7%, 1%, 3%, and 5% (*w*/*v*). The pretreated lily scales, all obtained from a single batch, were randomly assigned to seven treatment groups. Each concentration consisted of three treatment replicates, with 20 individual scales per replicate (60 scales per concentration and 420 scales in total). The lily scales were completely immersed in the corresponding Na_2_S_2_O_5_ solution for 30 min to ensure sufficient contact between the samples and the treatment solution. After treatment, the samples were briefly rinsed with purified water to remove residual free bleaching agents from the surface and then drained. The treated samples were dried in a thermostatically controlled oven at 50 °C to constant weight. Each treatment replicate was treated independently in a separate container using a separately prepared aliquot of the corresponding treatment solution. The dried samples were subsequently subjected to hyperspectral image acquisition and SO_2_ residue determination.

### 2.2. Hyperspectral Data Acquisition

Hyperspectral images were acquired using a SOC710E hyperspectral imaging system (Surface Optics Corp., San Diego, CA, USA). The system consisted of a hyperspectral camera, an imaging lens, a sample stage, a multi-channel regulated DC power supply (MPS-3305, Shenzhen Maiwei Instrument Co., Ltd., Shenzhen, China), and two symmetrically positioned halogen lamps (220 V, 75 W, Philips, Shenzhen, China). The spectral range of the system was 370–1100 nm with a spectral resolution of 3 nm, and the integration time was set to 19 ms.

Prior to image acquisition, the hyperspectral imaging system and halogen light sources were preheated for 30 min to ensure illumination stability and measurement consistency. All hyperspectral images were collected in a darkroom to minimize interference from ambient light. The two halogen lamps were symmetrically positioned at an angle of approximately 45° relative to the sample surface, with a working distance of 30–35 cm, thereby providing uniform and stable illumination during image acquisition.

Before sample imaging, dark and reference images were acquired for relative-reflectance calibration of the raw hyperspectral images. The dark reference image was collected with the camera lens completely covered to eliminate incident light, whereas the reference image was obtained using a standard panel with a nominal reflectance of approximately 30%. The relative-reflectance image was calculated according to the following equation:

$$R=\frac{I-I_{dark}}{I_{ref}-I_{dark}}$$

where (*R*) is the calibrated relative-reflectance image, (*I*) is the raw hyperspectral image, (*I_dark_*) is the dark reference image, and (*I_ref_*) is the image of the 30% reflectance panel.

During image acquisition, the lily scales were evenly placed on the sample stage, ensuring that the sample surfaces were as flat as possible and did not overlap. Multiple scales could be imaged simultaneously in each acquisition, while an appropriate distance was maintained between samples to avoid overlap during subsequent region of interest (ROI) extraction. ROIs were manually delineated for each sample using ENVI (version 5.6) software, and the mean spectrum of all pixels within each ROI was extracted as the representative spectrum of the corresponding sample. Each scale therefore contributed one spectral observation. Accordingly, 20 spectral observations were obtained from each treatment replicate, yielding 60 spectral observations per concentration and 420 spectral observations in total.

### 2.3. Determination of SO_2_ Residues

SO_2_ residues were determined by the nitrogen-assisted distillation and acid-base titration procedure in GB 5009.34-2022 [24]. Each treatment replicate was ground and homogenized separately. A 35 g portion was acidified with hydrochloric acid during heating under continuous nitrogen flow; released SO_2_ was carried into hydrogen peroxide absorption solution and subsequently titrated with standardized 0.01 mol/L sodium hydroxide using methyl red as the indicator, with a reagent blank analyzed in parallel. Each treatment replicate was measured in analytical triplicate, and the analytical mean was assigned as its reference value. Under these conditions, the method detection and quantification limits were 1 and 10 mg/kg, respectively. All untreated-control replicates were below the detection limit and are reported as <1 mg/kg; zero was used only as a numerical modeling code. Thus, the study contained 21 independent chemical reference values, one for each concentration × treatment-replicate combination. For contextual comparison, GB 2760-2024 specifies a maximum level of 0.2 g/kg (200 mg/kg), expressed as SO_2_ residue, for food category 04.02.02.02 (dried vegetables) [25]. Because this value is specific to the designated food category, it was used only as a contextual reference in this study and not as a universal safety threshold or a direct criterion for model-based compliance determination.

### 2.4. Spectral Data Preprocessing and Model Validation

Hyperspectral data may be affected by instrumental noise, light scattering, baseline variation, sample-surface heterogeneity, and illumination differences. Therefore, seven spectral preprocessing strategies were evaluated, including no preprocessing (Raw), Savitzky–Golay smoothing (SG), multiplicative scatter correction (MSC), standard normal variate transformation (SNV), SG combined with MSC (SG + MSC), SG combined with SNV (SG + SNV), and normalization (NOR).

Savitzky–Golay smoothing used a 9-point window, a second-order polynomial, and derivative order 0 [26]. Smoothing was applied to the full acquired spectrum before restricting the analysis to 400–1000 nm. SNV was calculated separately for each spectrum, NOR used the L2 vector norm, and the MSC reference spectrum was estimated from inner-training data only. All preprocessing, centering, scaling, and model tuning were fitted within the corresponding calibration data and then applied unchanged to held-out samples.

A treatment-replicate-grouped nested three-fold validation design was used. In outer fold r, treatment replicate r was held out simultaneously across all seven concentrations, while the other two replicate sets formed the outer-calibration data. Within each outer-calibration set, the two available replicate sets alternated as inner-training and inner-validation sets. Candidate preprocessing-model pipelines and bounded hyperparameters were evaluated by mean inner-fold macro-F1 for classification or RMSE for regression. The selected setting was refitted on both outer-calibration replicate sets and used once to predict the outer held-out replicate set (Algorithm S1) [27].

Classification retained all 420 scale-level spectra, and pooled out-of-fold predictions were evaluated at the scale level. For regression, the 20 spectra belonging to each concentration × treatment replicate were averaged before model fitting, producing 21 independent mean-spectrum/reference pairs that matched the chemical measurement hierarchy. Regression R^2^, RMSE, MAE, and RPD were calculated from the 21 pooled outer-fold predictions. This design prevents the 20 scales sharing one homogenate reference value from being treated as 20 independent chemical measurements.

### 2.5. Development of Classification Models for Na_2_S_2_O_5_ Treatment Levels

RF, KNN, and radial-basis-function SVM were evaluated for seven-class treatment-level classification [19,20,21]. RF used 800 trees and bounded grids for mtry, terminal-node size (5, 10, or 20), and maximum nodes (20, 40, or 80). KNN evaluated odd k values from 5 to 25 after calibration-only centering and scaling. SVM evaluated cost values of 0.25, 0.5, 1, 2, 5, and 10 and gamma values of 0.25/p, 0.5/p, and 1/p, where p is the number of predictors. Hyperparameters were selected only within the inner treatment-replicate loop.

Classification performance was summarized by accuracy, Cohen’s kappa, balanced accuracy, macro-F1, row-normalized confusion matrices, and one-versus-rest AUC values calculated from the same pooled held-out predictions. Preprocessing and hyperparameters were fixed within each outer fold before its validation data were predicted.

### 2.6. Development of Regression Models for Treatment-Associated SO_2_ Residue Estimation

Three regression approaches were evaluated for treatment-associated SO_2_ residue estimation: RF, SVR, and PLSR. The regression response was the experimentally measured SO_2_ residue expressed in mg/kg as a continuous variable rather than the nominal Na_2_S_2_O_5_ treatment label. RF regression used 600 trees and optimized mtry within calibration folds. SVR used a radial basis function kernel; predictors were standardized using calibration-fold statistics only, while the response remained in mg/kg. The evaluated SVR grid included cost values of 1, 10, 100, and 1000, gamma values of 0.5/p, 1/p, and 2/p, and epsilon values of 2, 5, 10, 25, and 50 mg/kg. PLSR models evaluated up to 20 latent components, subject to the number of available observations and variables; to reduce unstable high-component solutions, component selection used a conservative one-standard-error criterion during validation. All preprocessing and hyperparameter choices were made using calibration data only within each validation fold [18,19,20,27,28].

The predictive performance of the regression models was assessed using the coefficient of determination (R^2^), root mean square error (RMSE), and residual predictive deviation (RPD). R^2^ was used to describe the proportion of variation in SO_2_ residue levels explained by the model, whereas RMSE was used to quantify prediction error in mg/kg. RPD was used as an additional indicator of predictive performance relative to the variation in the reference values. In general, higher R^2^ values, lower RMSE values, and larger RPD values indicate better predictive performance.

### 2.7. SHAP-Based Model Interpretability Analysis

SHAP analysis was conducted after cross-validated performance evaluation to interpret the wavelength contributions of the selected classification and regression pipelines. This analysis was used solely for model interpretation and did not affect model selection or performance estimation. Model-agnostic approximate permutation SHAP values were calculated using the R (version 4.5.0) package fastshap (version 0.1.1), with 30 Monte Carlo permutations, adjustment enabled (adjust = TRUE), and a fixed random seed of 20260814. For classification, 140 spectra were used as the background set, and 70 spectra—10 from each class—were explained. The explained output was the predicted probability assigned to each sample’s observed class. For regression, all 21 treatment-replicate mean spectra were used as both the available background pool and the explained units, and the explained output was the predicted treatment-associated SO_2_ residue. All wavelengths within the modeling range were retained in the fitted models. Mean absolute SHAP values were used only to rank wavelength contributions, and the 15 highest-ranked wavelengths were displayed without refitting the models using this subset. Bar plots, summary plots, and dependence plots were used to visualize global wavelength importance, the distributions of SHAP contributions, and wavelength–response relationships, respectively [22,23].

## 3. Results

### 3.1. Effects of Different Na_2_S_2_O_5_ Treatments on Appearance Characteristics, SO_2_ Residues, and Hyperspectral Responses of Lanzhou Lily

Na_2_S_2_O_5_ treatment produced clear concentration-dependent changes in the appearance and SO_2_ residue levels of Lanzhou lily. The untreated samples showed a natural pale-yellow color with visible heterogeneity, whereas treatment at 0.3–0.7% produced a brighter and more uniform appearance. Increasing the concentration to 1–5% further increased apparent whiteness, but the visual improvement became less pronounced at the highest treatment levels. These observations indicate that the bleaching effect increased with Na_2_S_2_O_5_ concentration, while excessive treatment provided progressively smaller additional gains in appearance quality.

**Figure 1:**
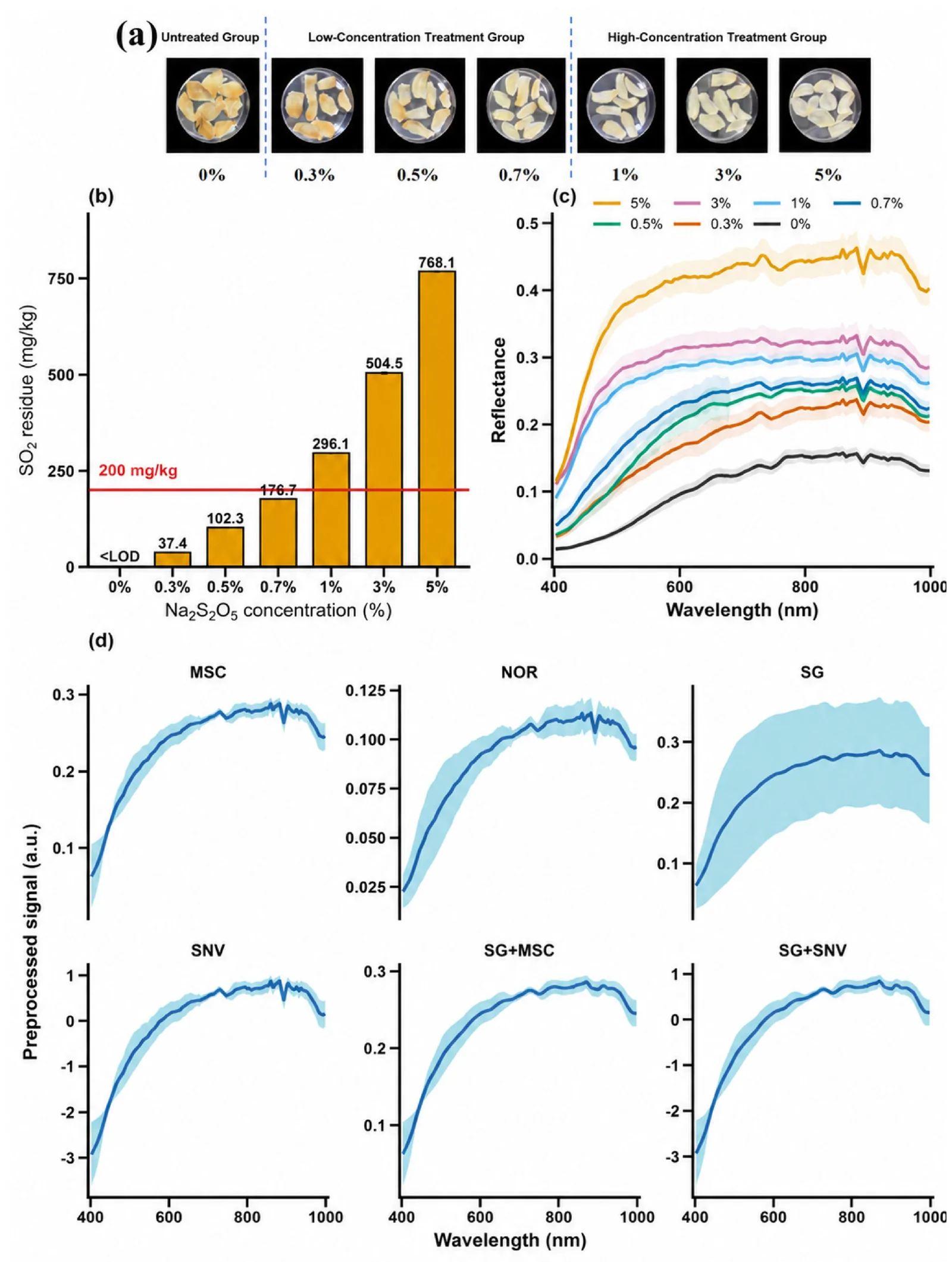
(**a**) Representative images of Lanzhou lily scales treated with different Na_2_S_2_O_5_ concentrations; (**b**) treatment-replicate mean SO_2_ residue with standard deviation (*n* = 3), with the untreated control shown as <LOD and the red line showing the category-specific 200 mg/kg contextual reference from GB 2760-2024,; (**c**) mean reflectance spectra and standard-deviation bands; (**d**) representative preprocessed spectra.

SO_2_ residues increased with Na_2_S_2_O_5_ concentration (Figure 1b). All untreated controls were below the 1 mg/kg detection limit. The 0.3%, 0.5%, 0.7%, 1%, 3%, and 5% groups contained 37.4 ± 0.6, 102.3 ± 0.5, 176.7 ± 0.5, 296.1 ± 0.9, 504.5 ± 2.9, and 768.1 ± 0.4 mg/kg, respectively (mean ± SD, *n* = 3 treatment replicates). The 0.3–0.7% groups were below the category-specific 200 mg/kg reference for dried vegetables, whereas the 1–5% groups exceeded it.

The mean reflectance spectra retained a broadly similar shape across all treatment groups but differed systematically in magnitude (Figure 1c). Within the 400–1000 nm modeling range, reflectance increased rapidly in the short-wave visible region and remained relatively high through the visible-near-infrared region. Higher Na_2_S_2_O_5_ concentrations generally produced higher reflectance, with the 5% group showing the highest overall response and the untreated control the lowest. The concentration-associated separation of the spectral curves indicates that the bleaching treatments generated reproducible optical differences suitable for subsequent classification and residue estimation.

The preprocessing methods produced distinctly different spectral representations (Figure 1d). SG reduced local high-frequency variation while preserving the overall spectral shape; MSC corrected multiplicative and additive scattering effects; SNV standardized each spectrum and consequently yielded both positive and negative transformed values; and NOR reduced variation in overall spectral magnitude. The combined SG + MSC and SG + SNV procedures reflected the sequential effects of smoothing followed by scatter correction or standardization. These differences support evaluating preprocessing together with the modeling algorithm rather than assuming that one preprocessing strategy is optimal for all tasks [1].

### 3.2. Classification Performance of Different Spectral Preprocessing Methods and Machine Learning Models for Identifying Na_2_S_2_O_5_ Treatment Levels

**Figure 2:**
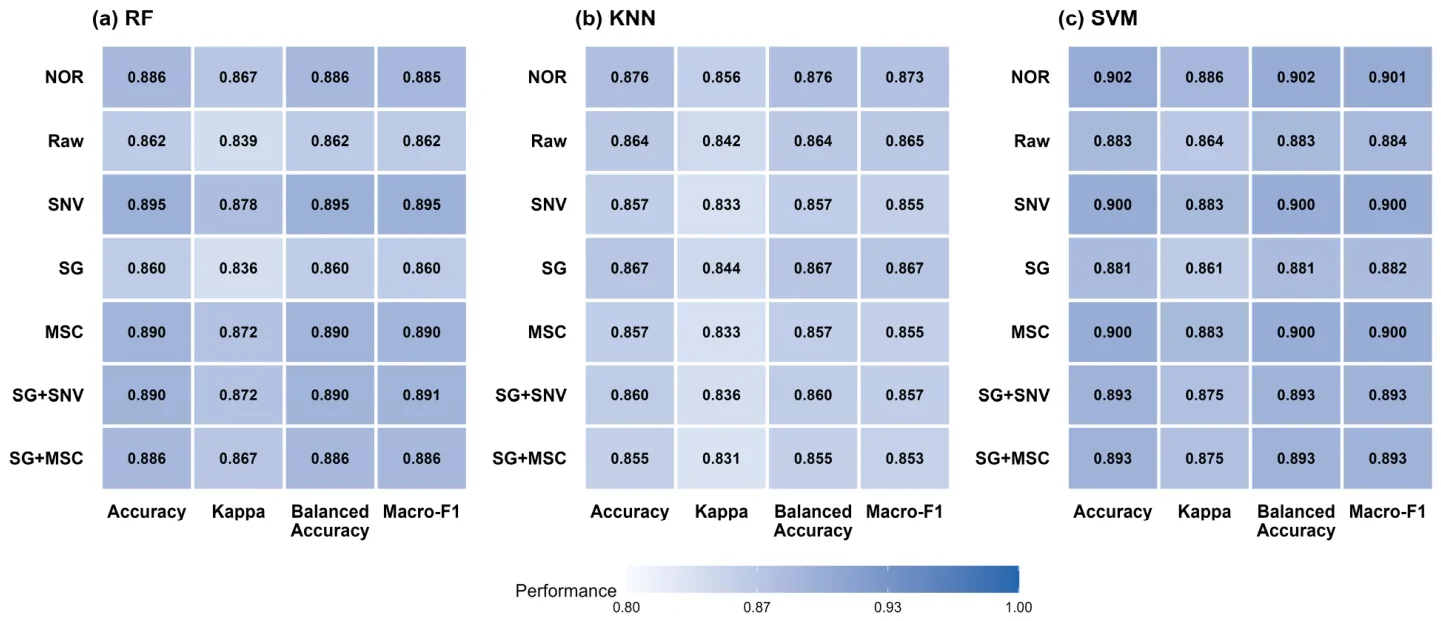
Validation performance of RF, KNN, and SVM classification models combined with different spectral preprocessing methods for identifying Na_2_S_2_O_5_ treatment levels in Lanzhou lily.

**Figure 3:**
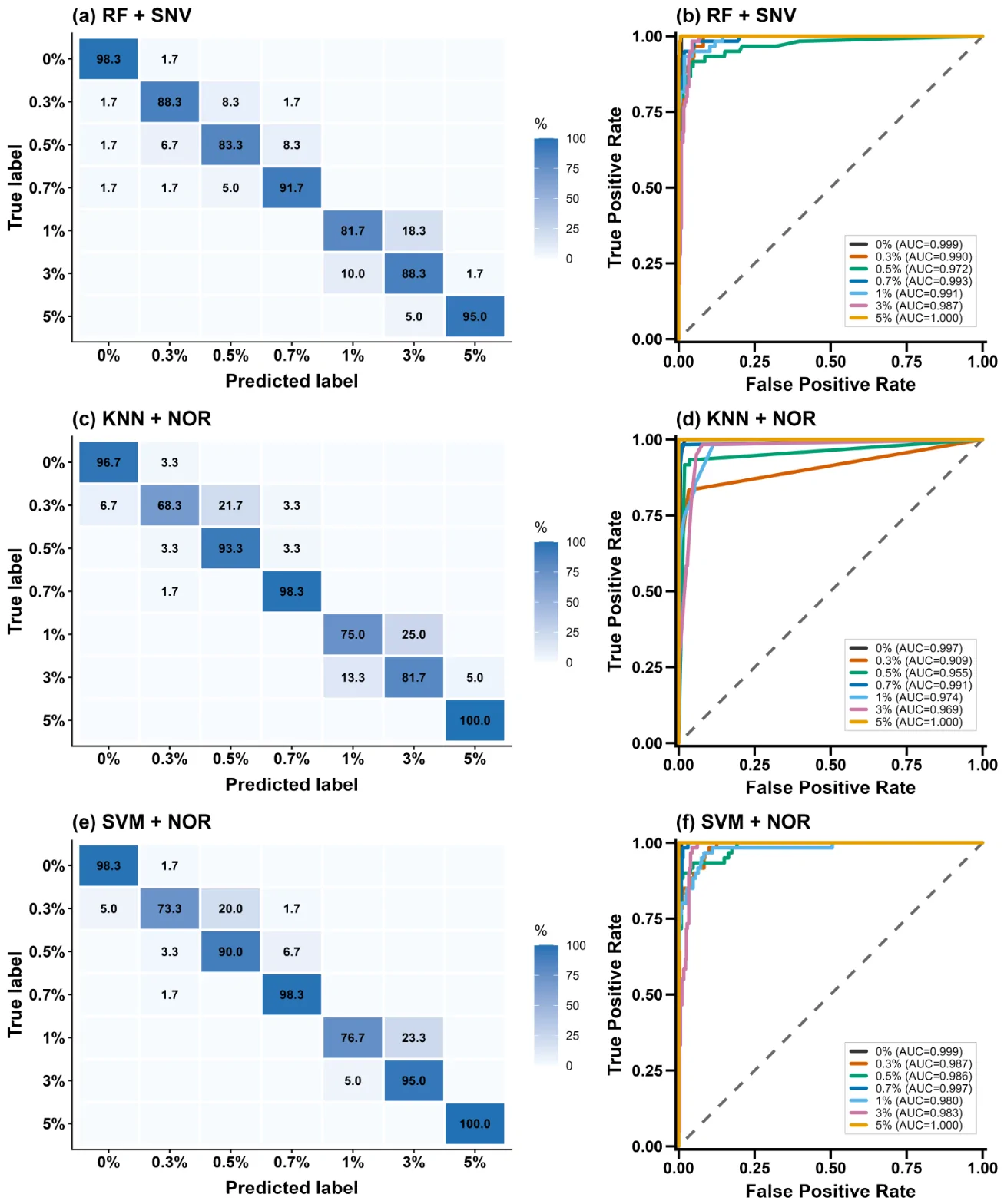
Out-of-fold classification results for the selected RF-SNV, KNN-NOR, and SVM-NOR pipelines. Panels (**a**,**c**,**e**) show row-normalized confusion matrices; panels (**b**,**d**,**f**) show corresponding one-versus-rest ROC curves.

Classification performance depended on both the learning algorithm and spectral preprocessing method (Figure 2 and Table S1). Among all evaluated pipelines, SVM-NOR achieved the highest overall performance, with an accuracy of 0.9024, kappa of 0.8861, balanced accuracy of 0.9024, and macro-F1 of 0.9007. RF-SNV was the best-performing RF pipeline (accuracy = 0.8952; macro-F1 = 0.8953), whereas KNN-NOR performed best among the KNN pipelines (accuracy = 0.8762; macro-F1 = 0.8735). Thus, SVM-NOR exceeded RF-SNV and KNN-NOR in accuracy by 0.72 and 2.62 percentage points, respectively. All reported metrics were calculated from the same pooled outer-fold predictions used to construct the confusion matrices and receiver operating characteristic (ROC) curves in Figure 3.

The confusion matrices further showed that the classification errors were concentrated primarily between adjacent treatment levels. For SVM-NOR, the class-specific recall values for the 0%, 0.3%, 0.5%, 0.7%, 1%, 3%, and 5% treatments were 98.3%, 73.3%, 90.0%, 98.3%, 76.7%, 95.0%, and 100.0%, respectively. The relatively lower recall for the 0.3% treatment mainly resulted from its misclassification as 0.5%, whereas part of the 1% treatment was classified as 3%. In contrast, the untreated, 0.7%, 3%, and 5% treatments were identified with comparatively high recall. Similar concentration-adjacent error patterns were observed for RF-SNV and KNN-NOR, although KNN-NOR showed greater confusion for the 0.3%, 1%, and 3% treatments.

The one-versus-rest ROC analysis was consistent with the confusion-matrix results. The class-specific area under the ROC curve (AUC) values ranged from 0.980 to 1.000 for SVM-NOR, compared with 0.972–1.000 for RF-SNV and 0.909–1.000 for KNN-NOR. In all three models, the 5% treatment produced an AUC of 1.000, indicating clear spectral separation of the highest treatment level. The comparatively lower recall observed for some intermediate treatments, despite their generally high AUC values, suggests that most classification uncertainty occurred near the decision boundaries between spectrally similar neighboring concentrations rather than across widely separated treatment levels.

### 3.3. Performance of Regression Models for Treatment-Associated SO_2_ Residue Estimation

**Figure 4:**
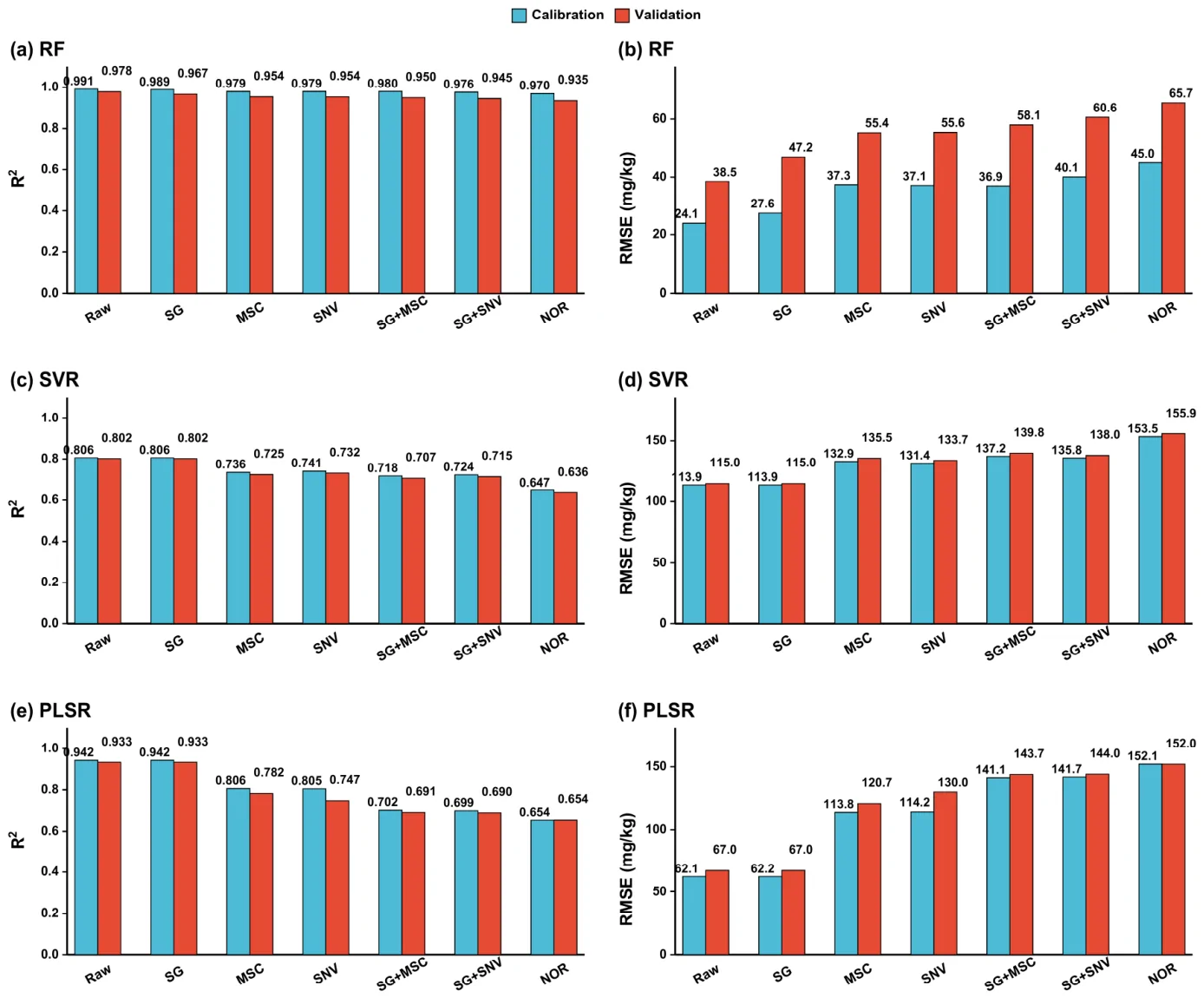
Comparison of calibration and validation performance for SO_2_ residue estimation using different combinations of preprocessing methods and regression algorithms. (**a**,**b**) RF; (**c**,**d**) SVR; (**e**,**f**) PLSR. R^2^ and RMSE are shown for each preprocessing strategy.

RF achieved the strongest regression performance among the evaluated algorithms (Figure 4 and Table S2). RF-Raw yielded a calibration R^2^ of 0.9913 and RMSE of 24.13 mg/kg, together with a treatment-replicate-grouped cross-validated R^2^ of 0.9778, RMSE of 38.47 mg/kg, MAE of 26.4 mg/kg, and RPD of 6.88. The R^2^ difference between calibration and cross-validation was 0.0134, and the cross-validation-to-calibration RMSE ratio was 1.59, indicating moderate performance attenuation during validation while retaining high predictive accuracy.

Preprocessing had different effects across the three algorithms. For RF, the raw spectra produced the lowest cross-validated RMSE, followed by SG (R^2^ = 0.9667; RMSE = 47.15 mg/kg). RF-MSC and RF-SNV showed similar performance, with R^2^ values of approximately 0.954 and RMSE values of approximately 55.5 mg/kg, whereas the combined preprocessing methods and NOR did not further improve the results. These findings indicated that the original reflectance profiles contained sufficient information for RF-based SO_2_ residue estimation. For SVR, Raw and SG produced the best and nearly identical results, with cross-validated R^2^ values of approximately 0.802 and RMSE values of approximately 115.0 mg/kg. PLSR performed better than SVR, with PLSR-Raw and PLSR-SG each achieving a cross-validated R^2^ of 0.9328 and RMSE of approximately 67.0 mg/kg. MSC, SNV, combined preprocessing, and NOR generally resulted in lower performance for both SVR and PLSR.

Comparison of the best pipeline from each algorithm showed that RF-Raw reduced cross-validated RMSE by approximately 42.6% relative to PLSR-Raw and by 66.5% relative to SVR-Raw. Accordingly, RF-Raw was selected as the final regression pipeline because it achieved the highest cross-validated R^2^ and the lowest RMSE and MAE among all evaluated model–preprocessing combinations.

**Figure 5:**
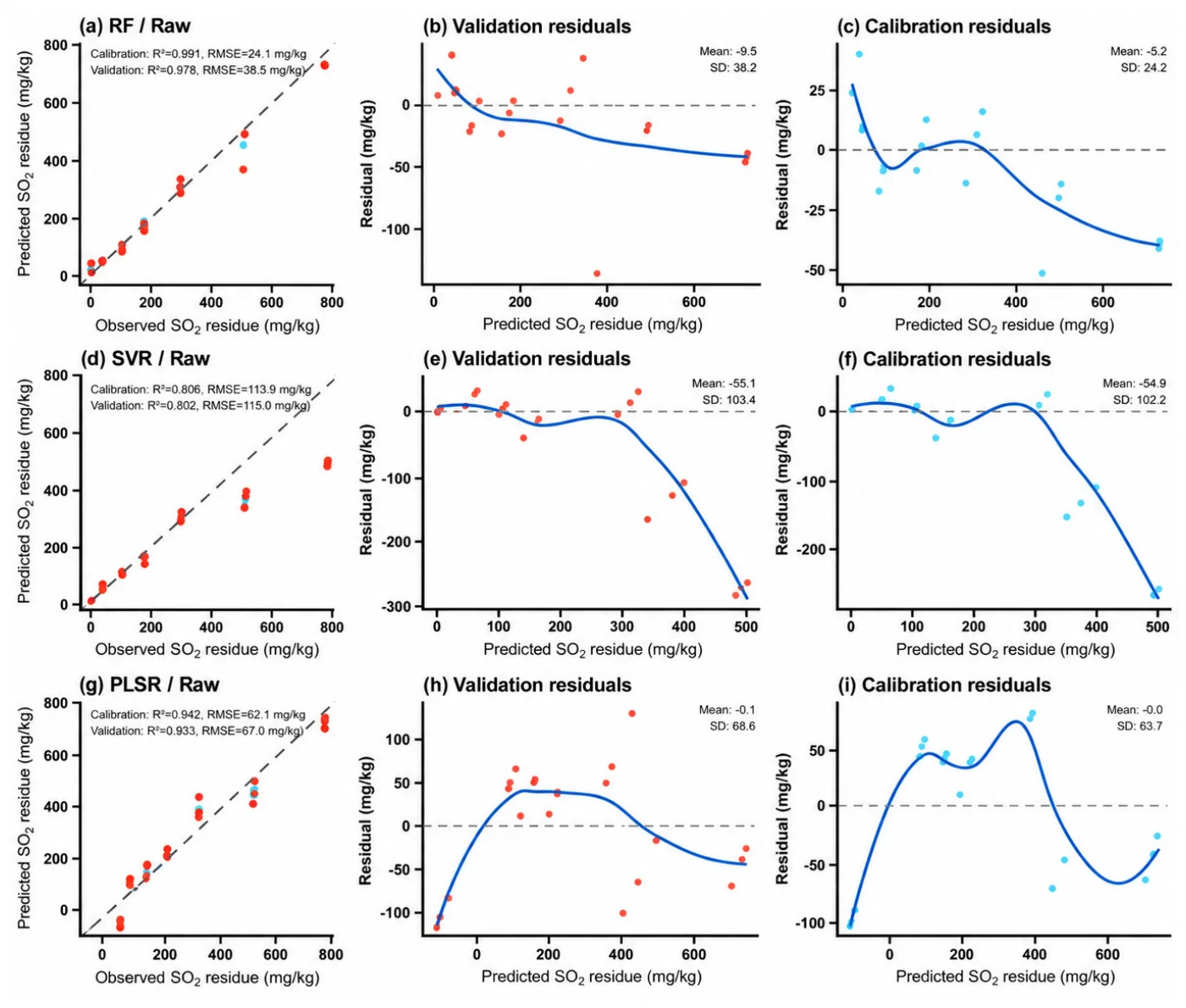
Measured-versus-predicted plots and residual diagnostics for the selected RF, SVR, and PLSR pipelines. Validation predictions and residuals are shown separately from calibration results.

The held-out R^2^/RMSE values for RF-Raw, SVR-Raw, and PLSR-Raw were 0.9778/38.5 mg/kg, 0.8020/115.0 mg/kg, and 0.9328/67.0 mg/kg, respectively (Figure 5). RF-Raw predictions were distributed closest to the 1:1 line and showed the lowest validation error, supporting its selection as the final regression pipeline. Although its validation residuals were more dispersed than its calibration residuals, the overall prediction pattern remained consistent with the performance metrics reported in Figure 4 and Table S2.

Supplementary analyses provided additional context for the model performance. The CIE L*, a*, and b*-only SVR baseline yielded R^2^ = 0.689 and RMSE = 144.1 mg/kg, showing poorer predictive performance than RF-Raw (Table S3 and Figure S2). The color-baseline method is described after Figure S2 in the Supplementary Materials. Because the algorithms and training units differed, this comparison does not isolate the contribution of additional spectral information. In the more stringent leave-one-treatment-level-out analysis, the pooled R^2^ and RMSE were 0.639 and 155.4 mg/kg, respectively; the RMSE values were 110.95 mg/kg when 0.7% was excluded and 173.4 mg/kg when 1% was excluded (Table S4A,B and Figure S1). These results indicate strong predictive performance within represented treatment levels but limited generalization to treatment concentrations absent from calibration.

### 3.4. SHAP Interpretation of the Selected Classification Model

**Figure 6:**
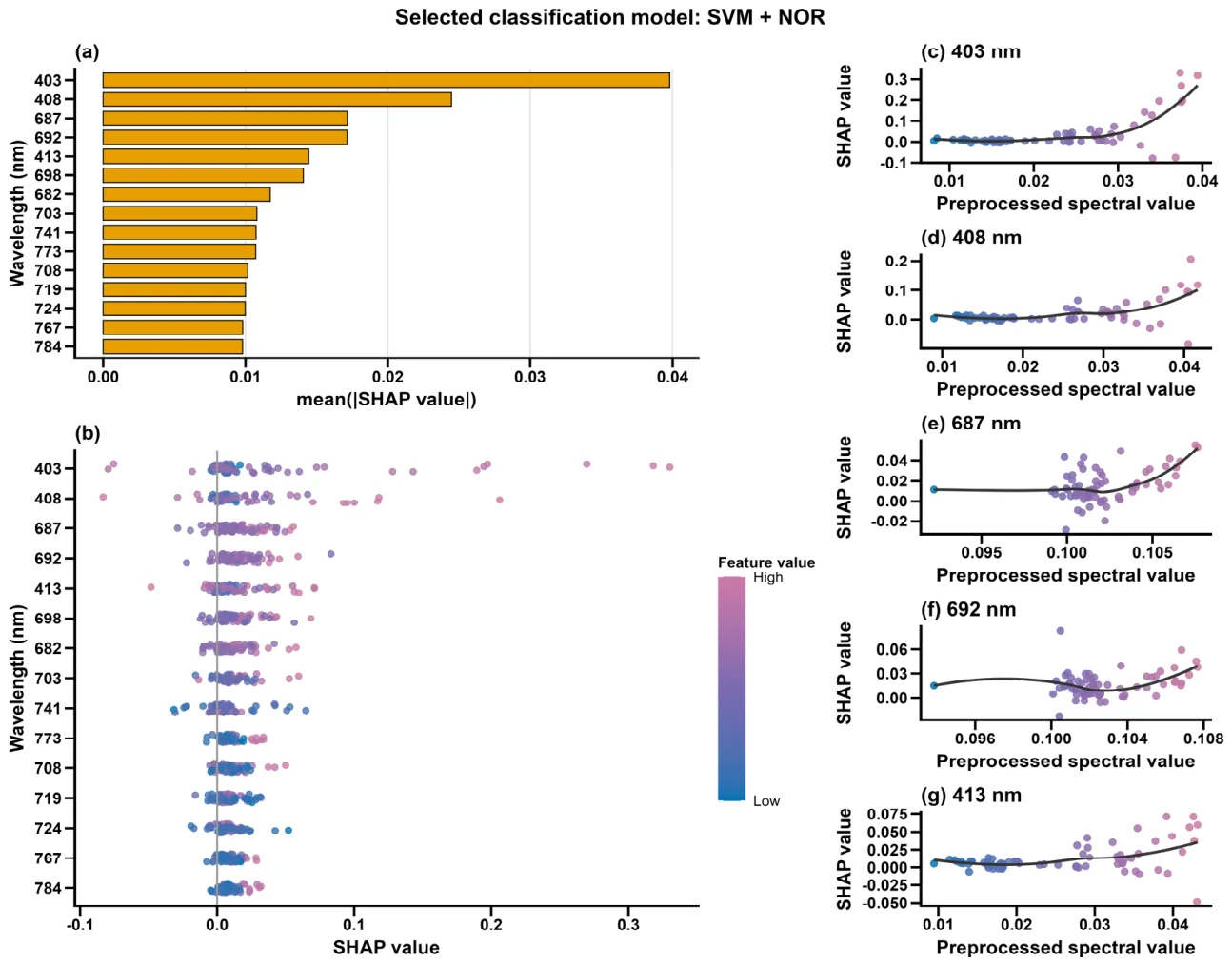
SHAP interpretation of the selected SVM-NOR classifier. (**a**) Mean absolute SHAP ranking; (**b**) summary plot; (**c**–**g**) dependence plots for the five highest-ranked wavelengths.

SHAP analysis identified 402.9, 407.9, 687.1, 692.4, and 413.0 nm as the five highest-ranked wavelengths for bleaching-level classification, with additional influential bands distributed mainly between approximately 681.8 and 783.6 nm (Figure 6 and Table S6). The clustering of adjacent wavelengths indicates that the classifier used correlated spectral regions rather than isolated individual bands. Overall, the selected model incorporated information from both the short-wavelength visible region and the red to near-infrared region.

In the spectral-edge sensitivity analysis, excluding the short-wavelength boundary region and repeating the complete validation reduced the accuracy of SVM-NOR from 0.902 to 0.874 (Table S5). This result indicates that the short-wavelength region contributed useful information but was not the sole basis of classification. Accordingly, the SHAP dependence plots represent the statistical contribution of these wavelength regions to the fitted classifier rather than direct evidence of specific Na_2_S_2_O_5_ or SO_2_ absorption bands.

### 3.5. SHAP Interpretation of the Selected Regression Model

**Figure 7:**
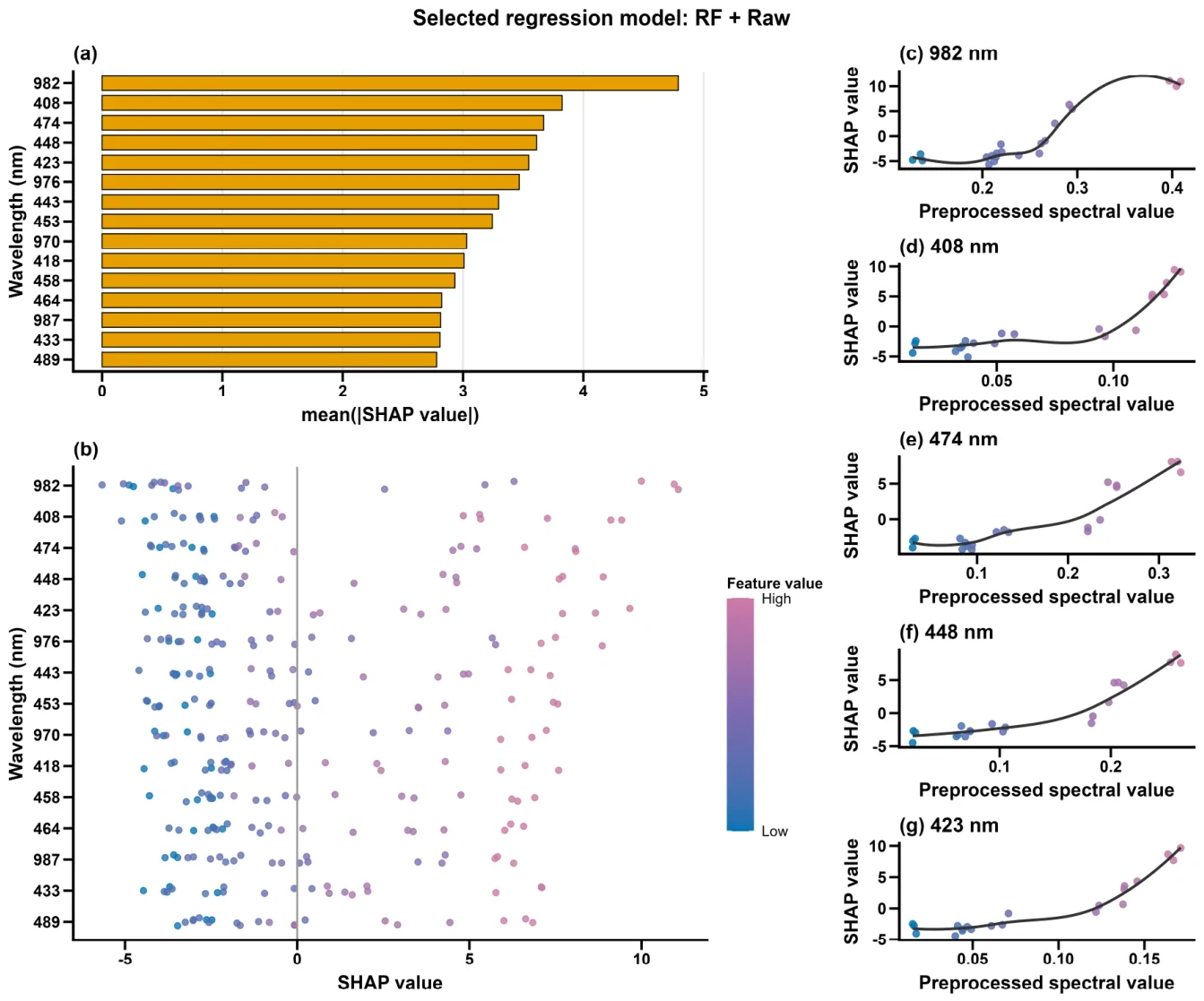
SHAP interpretation of the selected RF-Raw model for treatment-associated SO_2_ residue estimation. (**a**) Mean absolute SHAP ranking; (**b**) summary plot; (**c**–**g**) dependence plots for the five highest-ranked wavelengths.

For SO_2_ residue estimation, RF-Raw ranked 981.6, 407.9, 473.7, 448.3, and 423.0 nm as the five most influential wavelengths, followed by 976.0, 443.3, 453.4, 970.4, and 418.0 nm (Figure 7 and Table S7). The distribution of these wavelengths showed that the model combined information from the visible region with information near the long-wavelength boundary.

The spectral sensitivity analyses supported the stability of the main result. Removing the 885–905 nm region produced R^2^ = 0.971 and RMSE = 44.0 mg/kg, compared with R^2^ = 0.978 and RMSE = 38.5 mg/kg in the primary analysis. Excluding the short-wavelength edge yielded R^2^ = 0.968 and RMSE = 46.4 mg/kg (Table S5). Thus, RF-Raw retained strong predictive performance after either region was excluded, indicating that neither the systematic feature near 890–900 nm nor the short-wavelength boundary alone determined the model predictions.

The classification and regression models used overlapping but nonidentical spectral regions. SVM-NOR placed greater emphasis on the short-wavelength visible and red/near-infrared regions, whereas RF-Raw combined visible wavelengths with bands near the long-wavelength boundary. This difference reflects the task-dependent use of spectral information by the fitted models. Accordingly, the identified wavelengths are interpreted as model-associated predictors under the present experimental design rather than as direct SO_2_ absorption bands.

## 4. Discussion

This study demonstrated the feasibility of combining HSI with replicate-aware machine learning for seven-level Na_2_S_2_O_5_ treatment identification and treatment-associated SO_2_ residue estimation in Lanzhou lily. SVM-NOR achieved the best classification performance, whereas RF-Raw provided the most accurate regression results. The different optimal preprocessing strategies suggest that classification and regression relied on different aspects of the spectral information. Normalization improved the discrimination of treatment levels, whereas the original reflectance profiles were more suitable for RF-based residue estimation. This finding also indicates that intensive spectral correction does not necessarily improve model performance and may remove useful treatment-associated variation [29].

The classification findings are consistent with previous optical studies showing that sulfur treatment produces detectable spectral changes in herbal and food materials. Zhang et al. [30] reported high sensitivity and specificity for distinguishing sun-dried and sulfur-fumigated Chinese herbal materials. Zhang et al. [31] applied HSI and deep learning to the discrimination of fumigated lilies and obtained an R^2^ of 0.755 for SO_2_-content prediction. Direct numerical comparison between these studies is limited by differences in materials, treatment procedures, spectral ranges, reference measurements, and validation strategies. Nevertheless, the collective evidence supports the potential of HSI for rapid evaluation of sulfur-treated agricultural products.

From a food-safety perspective, the proposed approach is most appropriately positioned as a rapid prescreening tool. Sulfites are effective processing agents but may cause adverse reactions in susceptible consumers [6,7]. The 200 mg/kg reference presented in Figure 1 corresponds specifically to dried vegetables under GB 2760-2024 [25]. However, the replicate-grouped performance applies to treatment levels represented in calibration within a single procurement lot. The LOCO RMSE values of 111.0 and 173.4 mg/kg for the held-out 0.7% and 1% levels, respectively (Table S4B), exceeded the approximately 23 and 96 mg/kg distances of their mean reference residues from the 200 mg/kg reference. Predictions for residues between calibrated treatment levels may carry similarly large errors and may therefore place samples on the incorrect side of the reference. Confirmatory testing using the reference method specified in GB 5009.34-2022 should not be restricted to samples whose predicted residues are close to the reference, because predictions farther from it may also be inaccurate. The proposed prescreening use requires further validation with additional samples near the decision level and from independent lots.

The color-only baseline performed substantially worse than the full-spectrum model, indicating that the predictive information was not adequately represented by the three CIE color coordinates alone. In addition, the spectral sensitivity analyses showed that the principal model performance was retained after removal of the short-wave boundary or the 885–905 nm region. These findings support the contribution of multiple spectral regions rather than dependence on a single edge feature. However, the influential wavelengths identified by SHAP should be interpreted as model-associated predictors rather than direct SO_2_ absorption bands.

Further validation using independent production batches and a broader distribution of SO_2_ reference values would strengthen the practical applicability of the method. Particular attention should be given to samples close to regulatory or operational decision levels, storage-related changes, moisture variation, and model transfer between instruments. These extensions would facilitate the development of HSI from a controlled experimental approach into a robust screening method for the quality and safety evaluation of sulfur-treated agricultural products.

## 5. Conclusions

Replicate-aware reanalysis identified SVM-NOR as the best bleaching-level classifier (accuracy = 0.902) and RF-Raw as the best treatment-associated SO_2_ residue estimator (R^2^ = 0.978; RMSE = 38.5 mg/kg) within the represented levels of a single procurement lot. The full-spectrum model outperformed a three-coordinate color baseline, but LOCO analysis showed limited performance for concentrations absent from calibration. HSI should therefore be considered a rapid, nondestructive prescreening approach that may prioritize samples for confirmatory SO_2_ testing, not a stand-alone compliance method. Validation across independent lots, bulbs, storage histories, instruments, and denser reference concentrations near the regulatory level is required before food-safety deployment.

## Data Availability

The raw scale-level hyperspectral data, treatment-replicate SO_2_ reference measurements, fold assignments, analysis code, and supporting model outputs are available from the corresponding author upon reasonable request. The raw hyperspectral dataset and corresponding SO_2_ reference measurements have also been provided to the Editorial Office for review and record-keeping.

## Supplementary Materials

The following supporting information can be downloaded at: https://www.mdpi.com/article/10.3390/foods15203650/s1, Algorithm S1: Treatment-replicate-aware nested validation procedure; Table S1: Classification performance; Table S2: Calibration and treatment-replicate cross-validation performance of regression pipelines; Table S3 and Figure S2: Full-spectrum versus CIE L*, a*, and b*-only diagnostic comparison; Table S4A,B and Figure S1: Leave-one-treatment-level-out sensitivity analysis; Table S5: Spectral-edge and 885–905 nm sensitivity analyses; Tables S6 and S7: SHAP wavelength rankings.
