# Supplementary material for: Hyperspectral Imaging and Interpretable Machine Learning for Bleaching-Level Identification and Treatment-Associated SO2 Residue Estimation in Lanzhou Lily
Source: Foods. 2026 Oct 14;15(20):3650. doi: 10.3390/foods15203650 (PMC13703516; doi:10.3390/foods15203650)
Supplement: Supplementary file 1 [file foods-15-03650-s001.zip › foods-4600486-supplementary.pdf]

Supplementary Materials

Title:

Hyperspectral Imaging and Interpretable Machine Learning for Bleaching-Level Identification and Treatment-Associated SO_2 Residue Estimation in Lanzhou Lily

Authors:

Yueling Yan [1,a], Maomei Xie [1,a], Jinzhi Liu [1], Liyi Deng [1], Jie Qiu [1], Shuang Yang [1], Lei Nie [2], Zheng Li [1,3,4,5], Huaxin Zhou [1,3,4,5*], Haixia Wang [1,3,4,5*]

[a] Yueling Yan and Maomei Xie contributed equally to this work.

Affiliations of Authors:

[1] *College of Pharmaceutical Engineering of Traditional Chinese Medicine, Tianjin University of Traditional Chinese Medicine, Tianjin, 301617, China*

[2] *NMPA Key Laboratory for Technology Research and Evaluation of Drug Products, School of Pharmaceutical Sciences, Cheeloo College of Medicine, Shandong University, Jinan 250012, China*

[3] *Tianjin Key Laboratory of Intelligent and Green Pharmaceuticals for Traditional Chinese Medicine, Tianjin 301617, China*

[4] *Haihe Laboratory of Modern Chinese Medicine, Tianjin 301617, China*

[5] *State Key Laboratory of Chinese Medicine Modernization, Tianjin 301617, China*

[*]Correspondence Authors at:

College of Pharmaceutical Engineering of Traditional Chinese Medicine, Tianjin University of Traditional Chinese Medicine, Tianjin, 301617, China.

E-mail addresses: zhxtcm@tjutcm.edu.cn (H.X. Zhou), whxtcm@tjutcm.edu.cn (H.X. Wang).

Algorithm S1: Treatment-replicate-aware nested validation procedure;

Table S1: Classification performance;

Table S2: Calibration and treatment-replicate cross-validation performance of regression pipelines;

Table S3 and Figure S2: Full-spectrum versus CIE L*, a*, and b*-only diagnostic comparison;

Tables S4A–S4B and Figure S1: Leave-one-treatment-level-out sensitivity analysis;

Table S5: Spectral-edge and 885–905 nm sensitivity analyses;

Tables S6–S7: SHAP wavelength rankings.

Table S1. Treatment-replicate three-fold cross-validation performance of classification models combined with different preprocessing methods.

Preprocessing	Model	Accuracy	Kappa	Balanced Accuracy	Macro F1
NOR	KNN	0.8762	0.8556	0.8762	0.8735
SG	KNN	0.8667	0.8444	0.8667	0.8671
Raw	KNN	0.8643	0.8417	0.8643	0.8648
SG+SNV	KNN	0.8595	0.8361	0.8595	0.8574
MSC	KNN	0.8571	0.8333	0.8571	0.855
SNV	KNN	0.8571	0.8333	0.8571	0.855
SG+MSC	KNN	0.8548	0.8306	0.8548	0.8529
SNV	RF	0.8952	0.8778	0.8952	0.8953
MSC	RF	0.8905	0.8722	0.8905	0.8901
SG+SNV	RF	0.8905	0.8722	0.8905	0.8907
SG+MSC	RF	0.8857	0.8667	0.8857	0.8858
NOR	RF	0.8857	0.8667	0.8857	0.8851
Raw	RF	0.8619	0.8389	0.8619	0.8622
SG	RF	0.8595	0.8361	0.8595	0.8599
NOR	SVM	0.9024	0.8861	0.9024	0.9007
MSC	SVM	0.9	0.8833	0.9	0.9001
SNV	SVM	0.9	0.8833	0.9	0.8999
SG+MSC	SVM	0.8929	0.875	0.8929	0.8931
SG+SNV	SVM	0.8929	0.875	0.8929	0.8926
Raw	SVM	0.8833	0.8639	0.8833	0.884
SG	SVM	0.881	0.8611	0.881	0.8816

Table S2. Calibration and treatment-replicate cross-validation performance of regression models.

Preprocessing	Model	Calibration R^2	Calibration RMSE mg/kg	CV R^2	CV RMSE mg/kg	CV RPD	R^2 Gap	RMSE Ratio
Raw	PLSR	0.9422	62.13	0.9328	66.99	3.95	0.0094	1.08
SG	PLSR	0.9422	62.16	0.9328	66.99	3.95	0.0093	1.08
MSC	PLSR	0.8061	113.81	0.782	120.67	2.19	0.0241	1.06
SNV	PLSR	0.8049	114.17	0.7471	129.97	2.04	0.0578	1.14
SG+MSC	PLSR	0.7019	141.11	0.6909	143.69	1.84	0.011	1.02
SG+SNV	PLSR	0.6993	141.73	0.6896	143.99	1.84	0.0097	1.02
NOR	PLSR	0.6536	152.13	0.6541	152	1.74	-0.0006	1
Raw	RF	0.9913	24.13	0.9778	38.47	6.88	0.0134	1.59
SG	RF	0.9886	27.56	0.9667	47.15	5.62	0.0219	1.71
MSC	RF	0.9792	37.29	0.954	55.43	4.78	0.0252	1.49
SNV	RF	0.9794	37.06	0.9538	55.56	4.77	0.0256	1.5
SG+MSC	RF	0.9796	36.88	0.9495	58.06	4.56	0.0301	1.57
SG+SNV	RF	0.976	40.07	0.945	60.64	4.37	0.031	1.51
NOR	RF	0.9696	45.04	0.9355	65.65	4.03	0.0342	1.46
Raw	SVR	0.8059	113.86	0.802	115	2.3	0.0039	1.01
SG	SVR	0.8059	113.86	0.802	115	2.3	0.0039	1.01
SNV	SVR	0.7414	131.43	0.7322	133.75	1.98	0.0092	1.02
MSC	SVR	0.7356	132.89	0.7251	135.52	1.95	0.0105	1.02
SG+SNV	SVR	0.724	135.79	0.715	137.98	1.92	0.009	1.02
SG+MSC	SVR	0.7181	137.22	0.7074	139.8	1.89	0.0107	1.02
NOR	SVR	0.6473	153.49	0.636	155.93	1.7	0.0113	1.02

Table S3. Full-spectrum RF-Raw model compared with the spectrally derived CIE L*, a*, b*-only SVR baseline.

Predictor Set	Model	N independent reference units	CV R^2	CV RMSE mg/kg	CV MAE mg/kg	CV RPD	RMSE increase vs full spectrum mgkg
Full reflectance spectrum, Raw	RF	21	0.9778	38.4721	26.3749	6.884	0
Spectrally derived CIE L*, a*, b*	SVR	21	0.6891	144.1	96.9043	1.8379	105.63

Table S4A. Pooled leave-one-treatment-level-out sensitivity analysis.

Model	Preprocessing	Pooled LOCO R^2	Pooled LOCO RMSE mg/kg	Pooled LOCO MAE mg/kg	Pooled LOCO RPD
RF	Raw	0.6387	155.36	132.54	1.7047

Table S4B. Leave-one-treatment-level-out performance summarized by held-out concentration.

Held out concentration	N treatment replicates	Observed mean mg/kg	Observed SD mg/kg	Predicted mean mg/kg	Predicted SD mg/kg	Bias mg/kg	RMSE mg/kg	MAE mg/kg
0%	3	0	0	81.4304	0	81.4304	81.4304	81.4304
0.3%	3	37.4	0.5568	82.514	0.9452	45.114	45.1279	45.114
0.5%	3	102.3	0.4583	144.87	6.4935	42.5653	42.8769	42.5653
0.7%	3	176.7	0.4583	65.9592	7.8557	-110.74	110.95	110.74
1%	3	296.07	0.8737	468.55	22.9219	172.48	173.44	172.48
3%	3	504.5	2.8792	305.69	3.6376	-198.81	198.82	198.81
5%	3	768.1	0.4359	491.46	0	-276.64	276.64	276.64

Note: For the untreated group, 0 mg/kg is the numerical modeling code for the measured result <LOD (<1 mg/kg), not an exact chemical measurement of zero.

Table S5. Sensitivity to exclusion of the short-wave edge and the 885–905 nm feature.

Task	Analysis	Model	Preprocessing	Accuracy	Balanced Accuracy	Macro F1	R^2	RMSE mg/kg	MAE mg/kg	RPD
Classification	400-1000 nm primary	SVM	NOR	0.9024	0.9024	0.9007	NA	NA	NA	NA
Regression	400-1000 nm primary	RF	Raw	NA	NA	NA	0.9778	38.4721	26.3749	6.884
Classification	423-1000 nm; short-wave edge excluded	SVM	NOR	0.8738	0.8738	0.8724	NA	NA	NA	NA
Regression	423-1000 nm; short-wave edge excluded	RF	Raw	NA	NA	NA	0.9677	46.422	29.3508	5.7051
Classification	400-1000 nm excluding 885-905 nm	SVM	NOR	0.9024	0.9024	0.9006	NA	NA	NA	NA
Regression	400-1000 nm excluding 885-905 nm	RF	Raw	NA	NA	NA	0.971	44.041	30.421	6.0135

Table S6. Top 15 SHAP-ranked wavelengths for the selected SVM-NOR classifier.

Rank	Wavelength nm	MeanAbsSHAP	Feature	Model	Preprocessing
1	402.9	0.0398	W 402.896	SVM	NOR
2	407.92	0.0245	W 407.923	SVM	NOR
3	687.13	0.0172	W 687.128	SVM	NOR
4	692.44	0.0171	W 692.443	SVM	NOR
5	412.95	0.0144	W 412.955	SVM	NOR
6	697.76	0.0141	W 697.762	SVM	NOR
7	681.82	0.0117	W 681.819	SVM	NOR
8	703.09	0.0108	W 703.087	SVM	NOR
9	740.51	0.0107	W 740.506	SVM	NOR
10	772.78	0.0107	W 772.783	SVM	NOR
11	708.42	0.0102	W 708.417	SVM	NOR
12	719.09	0.01	W 719.092	SVM	NOR
13	724.44	0.01	W 724.438	SVM	NOR
14	767.39	0.0098	W 767.391	SVM	NOR
15	783.58	0.0098	W 783.584	SVM	NOR

Table S7. Top 15 SHAP-ranked wavelengths for the selected RF-Raw regression model.

Rank	Wavelength nm	MeanAbsSHAP	Feature	Model	Preprocessing
1	981.58	4.7893	W 981.577	RF	Raw
2	407.92	3.8234	W 407.923	RF	Raw
3	473.75	3.6698	W 473.746	RF	Raw
4	448.32	3.6108	W 448.325	RF	Raw
5	423.03	3.5464	W 423.034	RF	Raw
6	975.99	3.467	W 975.986	RF	Raw
7	443.26	3.2959	W 443.256	RF	Raw
8	453.4	3.2433	W 453.398	RF	Raw
9	970.4	3.03	W 970.400	RF	Raw
10	417.99	3.008	W 417.992	RF	Raw
11	458.48	2.9327	W 458.477	RF	Raw
12	463.56	2.8225	W 463.562	RF	Raw
13	987.17	2.8138	W 987.174	RF	Raw
14	433.13	2.8084	W 433.135	RF	Raw
15	489.06	2.7805	W 489.061	RF	Raw

Figure S1. Leave-one-treatment-level-out sensitivity analysis for RF-Raw. Each point represents one independent treatment-replicate reference unit.

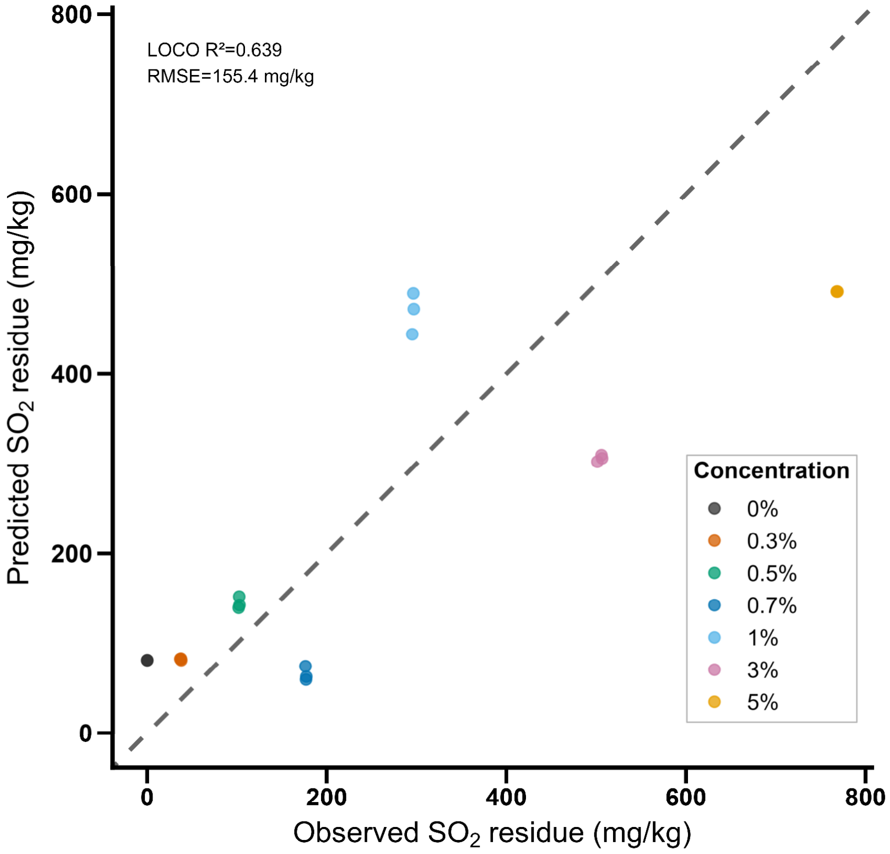

Figure S2. Treatment-replicate observed-versus-predicted values for (a) full-spectrum RF-Raw and (b) the spectrally derived CIE L*, a*, b*-only SVR baseline.

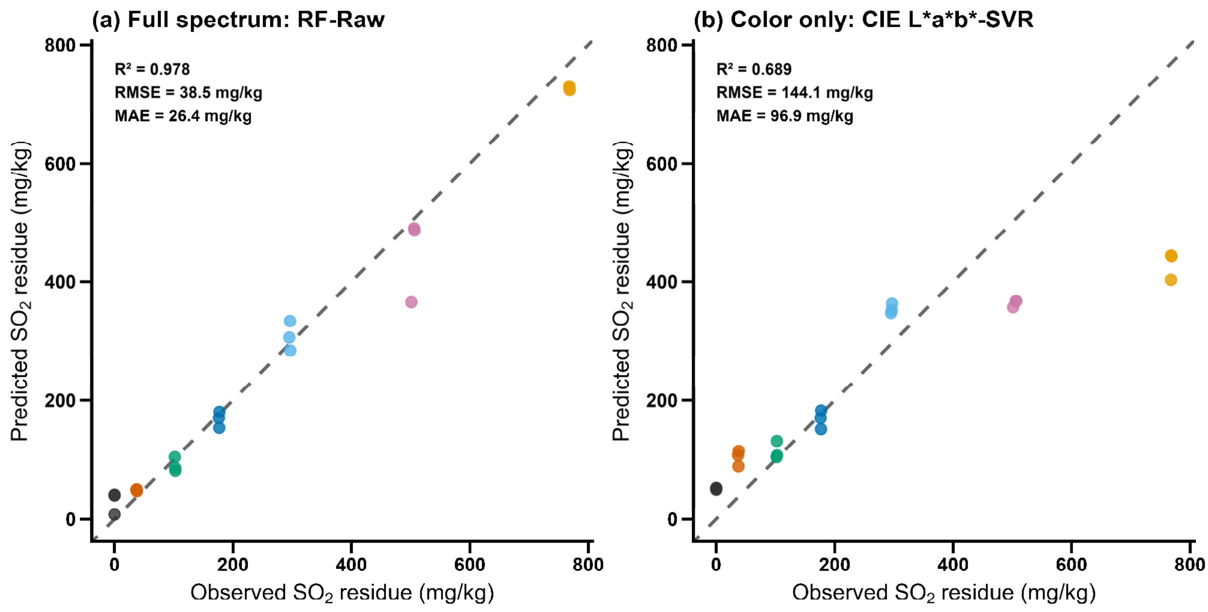

CIE L*a*b* Color-Baseline Analysis

CIE L*, a*, and b* coordinates were derived from the hyperspectral reflectance spectra and used to develop a color-only support vector regression (SVR) baseline with a radial basis function kernel. The model used the same treatment-replicate fold assignments as the full-spectrum analysis, with scaling and hyperparameter tuning performed only within the corresponding calibration data. Held-out scale-level predictions were averaged within each treatment replicate, and performance metrics were calculated from the 21 treatment-replicate observed–predicted pairs. This diagnostic analysis assessed the predictive capacity of three conventional color coordinates. Because its training unit and algorithm differed from those of the full-spectrum model, performance differences cannot be attributed solely to additional spectral information or taken as evidence of SO_2-specific detection.

Algorithm S1. Treatment-replicate-aware nested validation procedure

Input:

Spectra from seven treatment levels, with three treatment replicates per level and 20 scales per replicate;

one SO_2 reference value per treatment replicate;

predefined preprocessing–model pipelines and hyperparameter grids.

Prepare task-specific data:

Classification: retain the 420 scale-level spectra and their treatment-level labels.

Regression: average the 20 spectra within each treatment replicate to obtain 21 mean-spectrum/reference pairs.

For each predefined preprocessing–model pipeline:

For outer fold r = 1, 2, 3:

1. Hold out replicate set r across all seven treatment levels. Use the remaining two replicate sets for calibration.
2. Evaluate candidate hyperparameter settings by two-fold inner validation:

   Train on one calibration replicate set and validate on the other; then reverse their roles.

   Within each inner fold:

   Estimate any data-dependent transformation parameters using the inner-training data only.

   Apply the transformations to the training and validation data, fit the candidate model, and predict the inner-validation data.

   Calculate the mean inner-fold macro-F1 for classification or RMSE for regression.
3. Select hyperparameters using the inner-validation results:

   Classification: maximize mean macro-F1.

   Regression: minimize mean RMSE.

   For PLSR, apply the one-standard-error rule described in Section 2.6 for component selection.
4. Re-estimate any data-dependent transformation parameters and refit the model using all outer-calibration data.
5. Apply the fitted pipeline to the outer-validation data and store the held-out predictions.

End outer fold.

Pool predictions from the three outer folds:

Classification: evaluate the 420 scale-level predictions.

Regression: evaluate the 21 treatment-replicate predictions.

End pipeline.

Output:

Classification: accuracy, Cohen’s kappa, balanced accuracy,
macro-F1, confusion matrices, and
one-versus-rest AUC values.

Regression: R^2, RMSE, MAE, and RPD.

Compare the predefined pipelines using their pooled outer-fold results.
